# Management of thyroid disorders during the COVID-19 outbreak: a position statement from the Thyroid Department of the Brazilian Society of Endocrinology and Metabolism (SBEM)

**DOI:** 10.20945/2359-3997000000352

**Published:** 2021-04-12

**Authors:** João Roberto M. Martins, Danilo G. P. Villagelin, Gisah A. Carvalho, Fernanda Vaisman, Patrícia F. S. Teixeira, Rafael S. Scheffel, José A. Sgarbi

**Affiliations:** 1 Sociedade Brasileira de Endocrinologia e Metabologia Departamento de Tireoide Rio de Janeiro RJ Brasil Departamento de Tireoide, Sociedade Brasileira de Endocrinologia e Metabologia, Rio de Janeiro, RJ, Brasil.; 2 Universidade Federal de São Paulo Escola Paulista de Medicina Departamento de Medicina São Paulo SP Brasil Laboratório de Endocrinologia Molecular e Translacional, Disciplina de Endocrinologia e Metabologia, Departamento de Medicina, Escola Paulista de Medicina, Universidade Federal de São Paulo (Unifesp/EPM), São Paulo, SP, Brasil.; 3 Hospital da PUC-Campinas Endocrinologia e Metabolismo Campinas SP Brasil Endocrinologia e Metabolismo, Hospital da PUC-Campinas, Campinas, SP, Brasil.; 4 Unicamp Pós-graduação em Clínica Médica Campinas SP Brasil Pós-graduação em Clínica Médica, Unicamp, Campinas, SP, Brasil.; 5 Hospital de Clínicas da Universidade Federal do Paraná Departamento de Endocrinologia e Metabologia Curitiba PR Brasil Departamento de Endocrinologia e Metabologia (SEMPR), Hospital de Clínicas da Universidade Federal do Paraná (HC-UFPR), Curitiba, PR, Brasil.; 6 Instituto Nacional de Câncer José Alencar Gomes da Silva Unidade de Endocrinologia Oncológica Rio de Janeiro RJ Brasil Unidade de Endocrinologia Oncológica, Instituto Nacional de Câncer José Alencar Gomes da Silva (INCA), Rio de Janeiro, RJ, Brasil.; 7 Universidade Federal do Rio de Janeiro Faculdade de Medicina Rio de Janeiro RJ Brasil Faculdade de Medicina, Universidade Federal do Rio de Janeiro (UFRJ), Rio de Janeiro, RJ, Brasil.; 8 Universidade Federal do Rio Grande do Sul Faculdade de Medicina Hospital de Clínicas de Porto Alegre Porto Alegre RS Brasil Unidade de Tireoide, Hospital de Clínicas de Porto Alegre, Faculdade de Medicina, Universidade Federal do Rio Grande do Sul, Porto Alegre, RS, Brasil.; 9 Faculdade de Medicina de Marília Departamento de Medicina Disciplina de Endocrinologia e Metabolismo Marília SP Brasil Unidade de Tireoide, Disciplina de Endocrinologia e Metabolismo, Departamento de Medicina, Faculdade de Medicina de Marília, Marília, SP, Brasil.

**Keywords:** Thyroid disorders, hypothyroidism, hyperthyroidism, subacute thyroiditis, COVID-19

## Abstract

This position statement was prepared to guide endocrinologists on the best approach to managing thyroid disorders during the coronavirus disease (COVID-19) pandemic. The most frequent thyroid hormonal findings in patients with COVID-19, particularly in individuals with severe disease, are similar to those present in the non-thyroidal illness syndrome and require no intervention. Subacute thyroiditis has also been reported during COVID-19 infection. Diagnosis and treatment of hypothyroidism during the COVID-19 pandemic may follow usual practice; however, should avoid frequent laboratory tests in patients with previous controlled disease. Well-controlled hypo and hyperthyroidism are not associated with an increased risk of COVID-19 infection or severity. Newly diagnosed hyperthyroidism during the pandemic should be preferably treated with antithyroid drugs (ATDs), bearing in mind the possibility of rare side effects with these medications, particularly agranulocytosis, which requires immediate intervention. Definitive treatment of hyperthyroidism (radioiodine therapy or surgery) may be considered in those cases that protective protocols can be followed to avoid COVID-19 contamination or once the pandemic is over. In patients with moderate Graves’ ophthalmopathy (GO) not at risk of visual loss, glucocorticoids at immunosuppressive doses should be avoided, while in those with severe GO without COVID-19 and at risk of vision loss, intravenous glucocorticoid is the therapeutic choice. Considering that most of the thyroid cancer cases are low risk and associated with an excellent prognosis, surgical procedures could and should be postponed safely during the pandemic period. Additionally, when indicated, radioiodine therapy could also be safely postponed as long as it is possible.

## INTRODUCTION

The novel coronavirus disease (COVID-19) caused by the acute respiratory syndrome coronavirus 2 (SARS-CoV-2) has affected millions of people worldwide since the first reported case in December 2019 (1). In early 2020, Brazil became the epicenter of the outbreak in Latin America (2) and the second country with the highest infection rate worldwide, behind only the United States (3).

The COVID-19 pandemic requires a joint effort from health care professionals of all areas of knowledge, including endocrinologists, to help fight the progress and consequences of the infection within their areas of expertise (4–6). Increasing evidence suggests that patients with prior endocrine diseases are at increased risk of developing severe COVID-19 (7–9), especially individuals with type 2 diabetes mellitus (10,11) or obesity (12), while data on thyroid involvement in SARS-CoV-2 infection are still scarce (13–16). A few studies that have addressed this area of concern have found associations of COVID-19 infection with abnormalities of the pituitary-thyroid axis (17), subacute thyroiditis (18–21), Hashimoto’s thyroiditis (22), and thyrotoxicosis (23). Thus, the challenges for endocrinologists and clinicians caring for patients with COVID-19 are the recognition of potential thyroid abnormalities in patients with no preexisting thyroid disease and the management of patients with previously diagnosed thyroid disorder. Based on these considerations, the Thyroid Department of the Brazilian Society of Endocrinology and Metabolism has prepared the present position statement to guide endocrinologists on delivering the best care to patients with thyroid disorders during the pandemic. For preparation of this statement, the authors performed a search of the English language literature on PubMed, Google Scholar, SciELO, and LILACS using the keywords “COVID-19” plus “thyroid”, “hypothyroidism”, “hyperthyroidism”, “Graves’ disease”, Graves’ ophthalmopathy”, or “thyroiditis”.

## PATIENTS WITHOUT PREEXISTING THYROID DISEASE

Thyroid diseases are among the most common endocrine disorders in the general population and are highly prevalent in Brazil, one of the countries with the highest rates of thyroid diseases worldwide (24). About 12.3–17.5% of the adult population (25–27) has some type of thyroid disorder, many of whom are undiagnosed. Therefore, many individuals who become infected with COVID-19 may present with an unknown thyroid disorder.

Both SARS-CoV-1 and now SARS-CoV-2 have been associated with abnormal thyroid function (9). A retrospective Chinese study (17) identified lower serum TSH and total T3 levels in hospitalized patients with COVID-19 compared with controls not infected with the virus. These hormonal abnormalities affected 18% of 50 patients with laboratory-confirmed COVID-19 and worsened with the severity of the disease. Similar findings are generally observed in critically ill patients during the acute phase of several diseases in the absence of hypothalamic-pituitary-thyroid primary dysfunction, a condition known as “euthyroid sick syndrome” or “non-thyroidal illness syndrome” (28), and have also been reported in patients with nonsevere COVID-19 (29). However, the Chinese study reported that 34% of the patients with COVID-19 infection presented low serum TSH levels only (and normal T3), which is an uncommon finding in non-thyroidal illness syndrome (17).

In another retrospective study (23), including 287 consecutive patients hospitalized for COVID-19 in non-intensive care units, 20.2% had thyrotoxicosis, 5.2% had hypothyroidism, and 74.6% were euthyroid. In that study, Lania and cols. did not clarify the exact mechanisms responsible for thyroid dysfunctions. However, they hypothesized that thyrotoxicosis was caused by destructive thyroiditis since thyrotoxicosis was often mild, improved spontaneously, and antithyroid antibodies were negative in the nine patients in whom these antibodies were evaluated. More recently, Muller and cols. also showed a substantial number of thyrotoxicosis cases (n=13) among 85 patients with COVID-19 (30).

Subacute thyroiditis after COVID-19 has also been reported. The first case was reported in an 18-year-old woman in Italy, who presented typical manifestations of subacute thyroiditis 15 days after the identification of SARS-CoV-2 on oropharyngeal swab (20). The patient presented mild elevation in serum free T4 and free T3 levels, undetectable serum TSH level, and multiple diffuse hypoechoic areas on thyroid ultrasound. She was treated with prednisone 25 mg/day, and her thyroid function and inflammatory markers normalized within 40 days. After the publication of this case, other similar cases have been reported (18,19,21,31). Another recent report was the first description of a patient developing Hashimoto’s thyroiditis after (7 days) mild COVID-19 infection (22).

Multiple mechanisms could be involved and partially explain the thyroid abnormalities observed during COVID-19 infection, including direct effects of the virus on the thyroid and pituitary cells, and indirect systemic effects by inflammatory cytokines (9,17,23,32). Of note, the angiotensin-converting enzyme 2 receptor, considered the gateway of SARS-CoV-2 entry into cells, is highly expressed in the thyroid gland (33), but detection of SARS-CoV-2 specifically in the thyroid tissue has not been reported to date.

The finding of low T3 levels in the context of non-thyroidal illness syndrome has been associated with increased mortality (28), but there is currently no consistent evidence showing a benefit of T3 administration in patients with the syndrome (34). Still, an ongoing phase II randomized, double-blind, placebo-controlled trial is investigating the potential impact of high-dose intravenous T3 on the recovery of critically ill patients with COVID-19 infection (35).

Regarding patients without preexisting thyroid disorders, this statement conclude that clinicians and endocrinologists should keep in mind the occurrence of possible thyroid disorders during and after COVID-19 infection. The most frequent findings related to thyroid hormone levels in patients with COVID-19, particularly in individuals with severe disease, are similar to those present in the non-thyroidal illness syndrome and require no intervention. As these manifestations are generally transient and do not reflect an actual thyroid abnormality, investigation of thyroid function during acute COVID-19 infection in critically ill patients should be avoided and only performed when a thyroid disorder is strongly suspected. Thyroid function could be assessed in patients recovering from COVID-19 infection depending on the context, *e.g.*, severe COVID-19 infection, personal or family history of autoimmune thyroid disease, and presence of thyroid-related symptoms. Subacute thyroiditis may develop simultaneously with COVID-19 or up to 6 weeks after the symptoms of COVID-19 have disappeared (18–21,31), and nonsteroidal antiinflammatory agents should be favored in this setting (36). If required, low-dose prednisone, preferably up to 20 mg/day (37) or even 25 mg/day (21), could be safely administered when patients fail to respond to nonsteroidal antiinflammatory agents (36).

## PATIENTS WITH PREEXISTING THYROID DISEASE

### Hypothyroidism

Hypothyroidism is the most common thyroid disorder, ranging in prevalence from 5–15% of the general population. Hypothyroidism may be caused by Hashimoto’s thyroiditis and may occur after radioiodine therapy or thyroidectomy. Independent from the etiology, hypothyroidism treatment consists of levothyroxine replacement to maintain normal serum TSH levels (38).

One study showed no association between hypothyroidism and a higher risk of infection and increased morbidity and mortality with COVID-19 (39). However, poorly controlled hypothyroidism may increase a patient’s risk of viral infection and complications (40).

Importantly, ongoing recommendations for diagnosing and treating hypothyroidism should be maintained during the COVID-19 pandemic (39). The usual levothyroxine dose should be maintained if a patient develops COVID-19 infection (41,42), and frequent blood test monitoring should be avoided, especially in patients receiving regular treatment. The same applies for patients recently diagnosed with hypothyroidism or in whom treatment has not been initiated yet. An exception should be made for patients who manifest symptoms of uncontrolled hypothyroidism, in whom serum TSH and free T4 levels should be measured (39,40); telemedicine is an option for adjustment of levothyroxine dose in these patients (43).

Levothyroxine is available at different commercial presentations and generic forms, and no shortage of levothyroxine has been reported during the pandemic (39,41,42). The patients should be instructed not to stock up on levothyroxine, so this medication remains available for everyone (40).

### Graves’ disease

Management of Graves’ disease (GD), the leading cause of hyperthyroidism (44,45), includes antithyroid drugs (ATDs), radioiodine therapy, or surgery. Radioiodine therapy and total thyroidectomy (TT) are considered definitive therapies and intend to render the patient hypothyroid, requiring lifelong levothyroxine replacement, thus preventing recurrence of hyperthyroidism (44,45).

The diagnosis of GD is based on the occurrence of clinical findings of hyperthyroidism associated with the presence of diffuse goiter, Graves’ ophthalmopathy (GO) (20–50% of the cases), and laboratory tests compatible with thyrotoxicosis (44,45). Finding of positive serum thyroid receptor antibodies (TRAb) helps to define an autoimmune involvement. Thyroid scintigraphy should be reserved for patients with an equivocal diagnosis and in those with nodular goiter and undetectable TRAb (44,45).

The treatment of hyperthyroidism caused by GD in the current phase of the COVID-19 pandemic can be divided into two scenarios: 1) treatment of patients with a prior diagnosis of GD and on regular treatment with ATD, and 2) treatment of patients with recently diagnosed GD who have not started therapy yet.

In the first scenario, and especially at the current stage of the pandemic when face-to-face consultations can be difficult, treatment with ATD should not be interrupted, as any relapse would require an urgent medical appointment and increase the risk of complications (*e.g.*, thyroid storm), which can be triggered by infections, including COVID-19 infection (45). Treatment with ATD is generally maintained for 12–24 months; after this period, the medication can be suspended. Alternatively, prolonged use of low-dose ATD may be considered, as it is safe and may increase the chance of GD remission (46,47). During the pandemic, telemedicine may be an alternative to manage patients with hyperthyroidism (43). If possible, definitive treatment of GD (radioiodine therapy or TT) may be carried out after the pandemic is over.

In the second scenario (patients with a recent diagnosis of GD), ATD should be the first therapeutic option due to possible restrictions regarding nuclear medicine or surgical treatment at this time. The rare but potential side effects of ATDs should be kept in mind, especially agranulocytosis (< 500 neutrophils/mL). This complication affects 0.3–0.5% of the patients using ATD and has a mortality rate of about 5%. It occurs more frequently in older patients, at the beginning of treatment, and with high doses of ATD, especially methimazole (45). Patients should be advised about the possibility of leukopenia and to seek immediate help for measurement of white blood cell count in the presence of fever, odynophagia, and flu-like symptoms. In patients with severe side effects (agranulocytosis, drug-induced hepatitis), ATDs should be suspended, and definitive treatment should be performed (45,47).

Periods of increased stress (*e.g.*, war, pandemic) tend to be associated with increased rates of autoimmune diseases (48,49). Considering that, patients currently experiencing GD remission may present recurrence of the disease, and an eventual increase in the number of new GD cases may occur. Clinicians must be attentive to this fact to promptly identify new cases and carry out early diagnosis and treatment.

### Thyroid storm

Thyroid storm is a life-threatening condition characterized by excessive thyroid hormone secretion or release that can lead to the collapse of several organs and eventual death (50,51). Treatment of thyroid storm consists of supportive measures, beta-blockers, ATDs, and glucocorticoids. Some issues related to thyroid storm must be highlighted, considering the current pandemic. The treatment of thyroid storm should be carried out in a hospital setting, which increases the risk of the patient becoming infected with SARS-CoV-2. Additionally, the use of glucocorticoids during thyroid storm, which had been initially avoided in patients with COVID-19 infection, may be incorporated in the therapeutic arsenal of this endocrine emergency, since recent studies has demonstrated a beneficial effect of corticosteroids on outcome of patients infected with coronavirus after hospitalization (52,53).

In theory, it is possible for COVID-19 infection to trigger thyroid storm in patients with poorly controlled hyperthyroidism or in those with undiagnosed hyperthyroidism. Furthermore, the challenges in obtaining adequate medical follow-up in the context of the COVID-19 pandemic may, eventually, favor the onset of thyroid storm.

Concerning ATDs, the American Thyroid Association (ATA) favors the use of propylthiouracil over methimazole during thyroid storm, since propylthiouracil inhibits T4 to T3 conversion in peripheral tissues, consequently reducing the effects of T3 on target tissues and leading to faster clinical improvement (36). However, reduced T4 to T3 conversion can also be achieved effectively with other measures, such as administration of glucocorticoids (300 mg hydrocortisone intravenous load, followed by 100 mg every 8 hours), high doses of propranolol, and solutions containing inorganic iodine (36). Importantly, iodine-containing solutions, if chosen, must be administered at least 1 hour after the first dose of the chosen ATD.

Surgical treatment should be performed in the rare circumstance of a patient not responding satisfactorily to ATDs, developing severe side effects associated with these agents, or being unable to undergo radioiodine therapy (*e.g.*, unavailability of radioiodine, pregnancy, lactation). In preparation for thyroidectomy in these situations, it is recommended the administration of solutions containing inorganic iodine (potassium iodide, Lugol’s solution, and iodinated contrasts) in addition to corticosteroids and beta-blockers (as previously described) (54).

### Graves’ ophthalmopathy

Up to 50% of the patients with GD have some degree of clinically manifested GO, the main extrathyroidal manifestation of GD (44,45,50,55). However, most cases of GO are classified as mild and present remission either spontaneously or with general measures such as control of hyperthyroidism, use of eye drops and lubricating gels, and cessation of smoking. However, about 3–5% of the cases of GO progress to moderate/severe and severe forms, requiring more aggressive therapies such as retroorbital radiotherapy, use of systemic corticosteroids, and even urgent orbital decompression (55).

Glucocorticoids on immunosuppressive doses are the treatment of choice in patients with moderate/severe GO without risk of visual loss but should be avoided during the COVID-19 pandemic; during this time, we recommend other alternative measures, such as retroorbital radiotherapy. A cumulative radiotherapy dose of 20 Gy over 10 days may result in antiinflammatory effects and improve diplopia (55).

In patients with moderate/severe GO using glucocorticoids at nonimmunosuppressive doses (*e.g.*, prednisone < 20 mg daily), the glucocorticoid may be maintained. Reinforcement of measures to prevent SARS-CoV-2 infection is essential in these patients. In case these patients become infected with the virus, the glucocorticoid should be reconsidered, depending on the progression of the patient’s clinical condition.

In patients with severe GO who are not infected with the SARS-CoV-2 and are at risk of visual loss (due to optic neuritis or corneal ulcer), the therapeutic choice is intravenous glucocorticoid despite a potential increased risk of COVID-19 infection resulting from the therapy. In cases defined as medical emergencies (*e.g.*, severe GO with a risk of visual loss), once treatment with intravenous glucocorticoid starts, personal protection, hygiene, and social distancing must be escalated to reduce the risk of SARS-CoV-2 infection. However, if these patients at risk of visual loss are infected with COVID-19 while using systemic glucocorticoids, the treatment should be reassessed continuously depending on the progression of the ophthalmopathy and the COVID-19 infection. Surgical procedures such as eyelid occlusion (for corneal ulcers) and orbital decompression could be considered (55).

Hospitalization rates for COVID-19 seem to be unaffected by the use of certain types of immunomodulators by patients with rheumatic diseases (56–58). However, recent reports have indicated that the use of rituximab (an anti-CD20 monoclonal antibody) could increase the risk of severe secondary infection in patients with COVID-19 infection (57,58). Based on that, we recommend against the use of rituximab for the treatment of GO during the COVID-19 pandemic.

### Toxic nodular goiter

Toxic nodular goiter is the second most common cause of hyperthyroidism in the general population (36,50). Unlike GD, which may progress with remission with drug therapy alone, definitive treatment is usually required in patients with toxic nodular goiter (36,50). For those situations in which nuclear medicine or surgical procedures are not available due to the pandemic, ATDs may be used to control hyperthyroidism until definitive treatment can be performed (59). If ATDs are used, the same precautions must be taken regarding attention to side effects, as described above.

### Amiodarone-induced thyrotoxicosis

Thyrotoxicosis secondary to use of amiodarone can occur either by increased production of thyroid hormones (type 1) or by destruction of the thyroid parenchyma (type 2). Correct identification of the type of thyrotoxicosis is essential since the treatment of each type is different: ATDs should be used in type 1, while glucocorticoids are used in type 2 (60,61).

Concerning type 2 thyrotoxicosis, initial use of prednisone 30 mg/day is recommended, followed by a gradual dose decrease over approximately 3 months (61). This dose of prednisone may be immunosuppressive, thus exposing the patient to an increased risk of COVID-19 infection, particularly to severe forms of this disease (62). If the administration of glucocorticoids becomes necessary, the patients should be instructed to avoid any situations or habits that may increase their risk of becoming infected. The glucocorticoid should be administered for the shortest possible time and be gradually tapered.

### Thyroid cancer

Issues regarding malignant disease management during the COVID-19 pandemic is still controversial. As known, the majority of thyroid cancer cases, especially those found incidentally, are low risk and associated with an excellent prognosis (63). Nickel and cols., recently suggested that those surgical procedures could and should be postponed safely during the pandemic period (64).

In more aggressive cases in which surgery cannot be postponed, such as anaplastic thyroid cancer or invasive tumors and bulky lymph node metastasis, surgery should be performed under a safety protocol, usually regarding pre-operative SARS-CoV-2 testing and avoiding visitors during the hospitalization period. Patients should also respect social distancing pre and postoperatively (65). Aguiar Junior and cols., from AC Camargo Cancer Center, São Paulo, showed that using a preventing protocol not only with RT-PCR test 2-3 days before, but also questionnaire with any suggestive COVID-19 symptoms and social isolation, oncology patients operated during the pandemic period had a low rate of newly diagnosed COVID-19 infection and complications. Of 540 patients included, 41 (7,6%) tested positive and surgery was postponed. Among the remaining 454 patients with a negative test that operated, no COVID-19-related symptoms or complications were observed during the in-hospital postoperative period, and no readmissions due to COVID-19 were identified (65).

In the follow-up of patients with thyroid cancer, those with excellent, indeterminate, and biochemical incomplete response to therapy should keep taking their levothyroxine pills regularly. Subclinical hyperthyroidism due to suppressive therapy, in the meantime, is not considered a risk factor for complications due to COVID-19 (63).

Patients with advanced thyroid cancer, with distant metastases (especially the lungs), or using specific cancer drugs, such as sorafenib, lenvatinib, or vandetanib, may be at greater risk for severe COVID-19, both by the extent of the disease and by possible adverse effects of medicines. These patients must be more careful, maintain social isolation, and follow all other measures already disclosed by the competent authorities to high-risk people.

Nowadays, radioiodine therapy (RAI) is not recommended for all differentiated thyroid cancer cases (63). Those low to intermediate-risk cases and with low postoperative thyroglobulin should be spared from RAI (63), which is even more important when the risk getting COVID-19 infection is enhanced by going out to get treated.

In cases in which RAI is indicated, especially for metastatic disease, RAI could be safely postponed with no impact on recurrence rates (66,67). So, the recommendation is to postpone as long as it is possible. On the other hand, the Brazilian Committee for Nuclear Energy has changed RAI recommendation during the COVID-19 pandemic. They have published a document that allows physicists to release patients with a higher radiation activity (>50 mCi) than was previously allowed, which made it possible to give up 100 mCi of RAI in some cases, with no need for hospitalization (68).

## CONCLUSION

To the best of our knowledge, controlled hypothyroidism and hyperthyroidism are not associated with an increased risk of COVID-19 infection, nor these conditions predispose the patient to more severe forms of the disease. Patients with hyperthyroidism should be treated with ATDs, although clinicians must be aware of the possibility of rare side effects with these medications, particularly agranulocytosis. Definitive treatment of hyperthyroidism (with radioiodine therapy or TT) should be postponed until the pandemic is over.

When necessary, corticosteroids should be used for the shortest period possible and preferably at low doses (*e.g.*, prednisone 20 mg daily). In situations in which this therapy is required (*e.g.*, severe GO requiring glucocorticoids at immunosuppressive doses), the clinician must consider the risks and benefits of the treatment.

Considering that most of thyroid cancer cases are low risk and associated with an excellent prognosis, surgical procedures could and should be postponed safely during the pandemic period. When indicated, RAI could also be safely postponed as long as it is possible.

The Thyroid Department of the Brazilian Society of Endocrinology and Metabolism supports the development of research for a better understanding of the role of the thyroid in the context of the risk and severity of COVID-19 infection, and the progression and recovery from this disease.
